# A cross-sectional study of evidence-based practice and its determinants among healthcare professionals in Northwest Ethiopia

**DOI:** 10.3389/fmed.2024.1460203

**Published:** 2024-10-21

**Authors:** Yideg Melkamu, Mulusew Andualem Asemahagn, Ayinengida Adamu Walle, Yawkal Tsega

**Affiliations:** ^1^Public Health Emergency Management, Guhalla District Health Office, Guhalla, Ethiopia; ^2^Department of Health Systems Management and Health Economics, School of Public Health, College of Medicine and Health Sciences, Bahir Dar University, Bahir Dar, Ethiopia; ^3^Department of Health System and Management, School of Public Health, College of Medicine and Health Sciences, Wollo University, Dessie, Ethiopia

**Keywords:** evidence-based practice, determinant factors, health professionals, primary hospitals, Central Gondar zone, Ethiopia

## Abstract

**Background:**

Evidence-based practice (EBP) is the integration of current best evidence with clinical expertise by considering patient preferences and values. Evidence based practice has not been well studied in Ethiopia. Therefore, this study aimed to assess EBP and its determinants among health professionals working at primary public hospitals in the Central Gondar zone, Northwest Ethiopia.

**Methods:**

A facility-based cross-sectional study was conducted on 422 health professionals. A simple random sampling technique was used to select the study participants. The data were entered into EpiData version 4.6 and exported to SPSS version 25 for analysis. The descriptive, bivariable, and multivariable logistic regression analysis were conducted. Adjusted Odds Ratio (AOR) with 95% confidence intervals and *p*-value <0.05 were used to assess association of explanatory variables with EBP and declare statistical significance, respectively.

**Result:**

About 44.1% (95%CI: 39, 50%) of healthcare professionals had good evidence-based practice. Educational status (AOR: 3.05, CI: 1.07–8.66), spare time (AOR: 1.90, CI: 1.09, 3.31), good knowledge (AOR: 7.95, CI: 4.83, 13.08), good skill (AOR: 2.39: CI: 1.27, 4.53), training (AOR: 2.13, CI: 1.26, 3.58), and internet access (AOR = 2.02: 95% CI: 1.25–3.27) were found to be significant predictors of evidence-based practice.

**Conclusion:**

This study revealed that EBP was low compared to national standards. Moreover, having good knowledge and skill about evidence-based practice, being trained, having spare time and internet access and upgrading educational status of health care professionals would enhance good evidence-based practice.

## Background

The term evidence-based practice was first developed in the field of medicine in the form of evidence-based medicine in the early 1990s, but as its use expanded to include other health disciplines, it became known as evidence-based practice (EBP) (1, 2). It is the integration of current best evidence with their clinical expertise to make decisions for a specific client by considering his or her preferences and values (3, 4). As such, it is a decision-making process for practice through the thorough, explicit, and judicious use of the best available evidence from multiple sources (2, 5–13).

Best available research is contextually relevant and best in quality, which includes empirical evidence from randomized controlled trials (RCT), evidence from other scientific methods such as descriptive and qualitative research and the use of information from case reports (14). Clinical expertise, means the knowledge and skill of professionals in their field of study (15). And patient preference and value refer to patient perspectives, beliefs, expectations, and goals for health and life that influence choices; these must all be weighed against the evidence (16).

EBP is most prominent in the United Kingdom, Canada and the United States, where outcomes measurement and effectiveness in public services are increasingly seen as important by governments and citizens in the settings of high-income countries (17).

Carrying out the process of EBP involves five steps, which include formulating the clinical question, acquiring evidence to answer the question, appraising the quality of evidence, applying best evidence to patient care, evaluating the outcome of the evidence (7, 18). Validating evidence indicates evaluating the quality of evidence before making decisions. The evidence is ranked in the hierarchy based on its likelihood of bias, after utilizing EBP, it is important to monitor and evaluate any changes in outcomes so that positive effects can be supported and negative ones remedied, finally positive outcomes should share with colleagues and other health care organizations via rounds, presentation and conferences (19).

Health systems try to improve the quality of health services by formulating valid guidelines and standards and using results of the research in clinical practice and also, comparing their performance with it (20).

Worldwide, EBP has been emphasized by the World Health Organization (WHO) and the European Commission to provide health services based on the best research (21). It is considered the gold standard of care, and as such, it is now an expectation of patients, regulatory agencies, and healthcare funders. However, the concept of EBP is still in its infancy in developing countries like Ethiopia (15, 22).

The increasing volume of research information, availability of sophisticated medical care, client expectation to get the best possible care, and the rising health care expenditure compel the governments around the globe to embrace EBP. Even though research supports that, EBP promotes high-value health care, there is poor utilization of research findings in health decision making, nursing practice, and policy formulation (10).

Documented evidences indicated that approximately 400,000 people die every year from preventable medical errors, and this is now a public health epidemic and the third leading cause of death in the United States. Due to the lack of utilization of evidence-based practice during patient care, about 20–25% of treatments may be unnecessary or even harmful to the patient (12). On the other hand, clients’ outcomes are at least improved by 28% when clinical care is based on the best evidence.

Moreover, the WHO stated that most research activities in Africa are linked to educational or academic institutions. Additionally, it is not known how much health-related research is being utilized to improve healthcare practice (13). Findings from the developed world implied that integrating EBP into routine primary care practice is practical and attainable (23).

To bring evidence-based practice onto the agenda at national, regional, and local levels, Ethiopia arranged a workshop on evidence-based healthcare for different health professionals in Addis Ababa (24), institute of medicine (IOM) set a goal that by 2020, 90% of clinical decisions will be supported by accurate, timely and up-to-date clinical information and will reflect the best available evidence to achieve the best patient outcomes (25), the introduction of Kigali declaration to support the development of evidence-based healthcare systems in which decisions made by healthcare practitioners, managers, policy makers (26).

Despite the effort underway, studies highlighted that health care in Ethiopia is not evidence-based; rather it relies on experience, tradition, and untested theories. Also, at all levels of the health systems, there is little culture of using research evidence during decision-making. To ensure that future healthcare users can be assured of receiving such care, healthcare professionals must effectively incorporate the necessary knowledge, skills and attitudes required for EBP into education programs (27).

Most frequently perceived barriers were lack of time, inadequate EBP knowledge, organizational resistance, heavy workloads, and lack of training and poor access to good quality research. In addition to this, there is a lack of collaboration between researchers and policymakers, lack of internet access, shortage of resources, and deficiency of strong studies on the efficiency of EBP in healthcare practice are factors of EBP (13, 28).

Even though evidence-based health care has been shown to be an efficient and much-needed practice worldwide, developing countries have more difficulties in the accessibility of existing evidence and medical resources than developed countries (29). In major clinical areas, such as hospitals, the findings of research studies are not often translated into actual clinical practice. As a result, many patients receive suboptimal care, and some of them suffer from serious and avoidable harm to their health (30).

**Figure 1:**
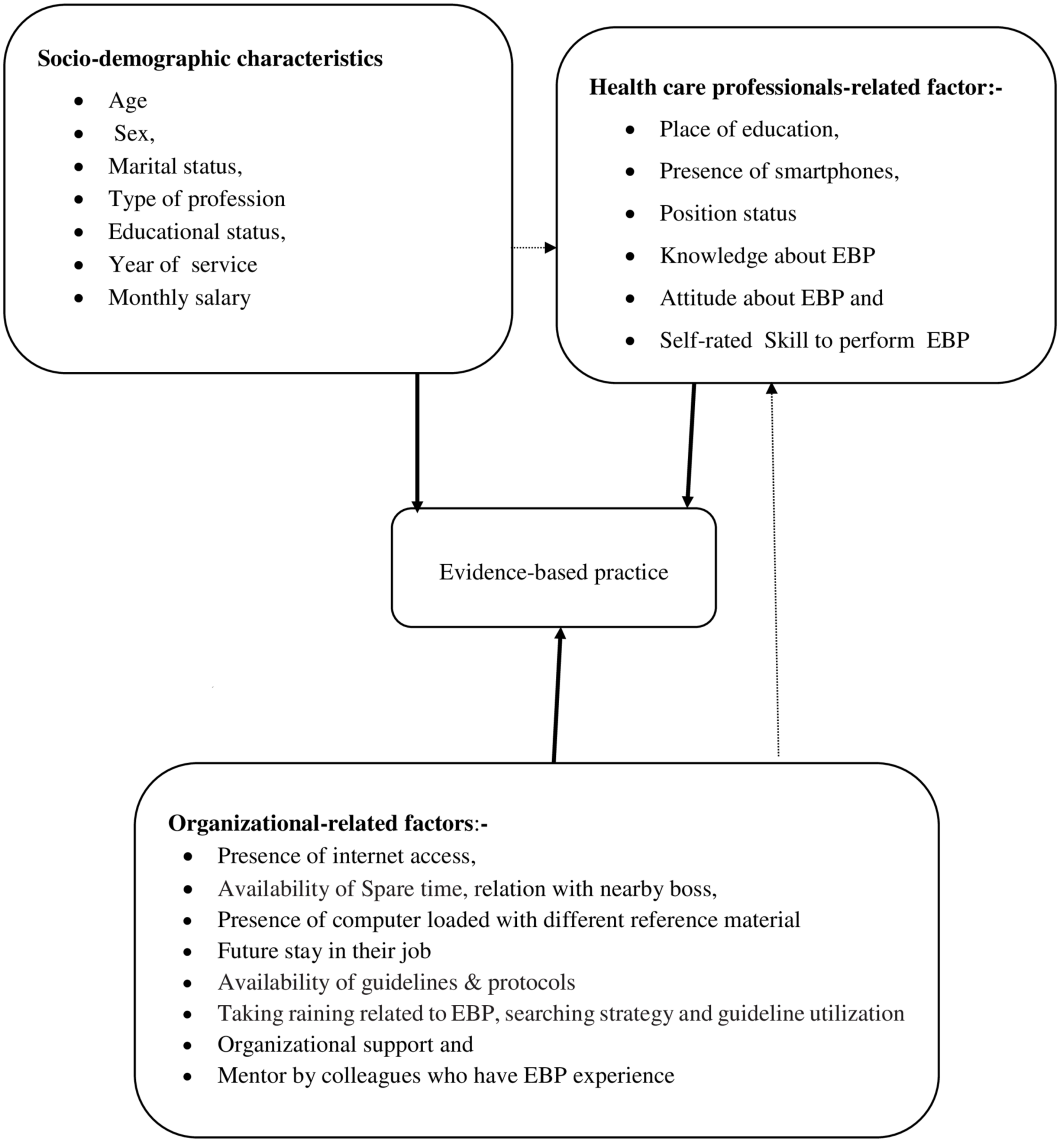
Conceptual framework of the study which is adapted from various studies, 2022.

Although there is a study conducted in general and referral hospitals, there is no study conducted in district-level hospitals. Therefore, this study aimed to assess EBP and its determinants among healthcare professionals working at public hospitals of Centeral Gondar zone (Figure 1).

## Methods

### Study setting, design, and period

This facility based cross-sectional study was conducted at public primary hospitals in the Central Gondar zone from 1st of May – 5th of June, 2022. This zone has 15 administrative districts, 375 Kebeles (338 rural and 37 urban), the smallest administrative unit in Ethiopia. Moreover, there are 9 public primary hospitals which consists a total of 875 health care professionals, 85 health centers, and 404 health posts with a total population of 2, 986,928 in Central Gondar zone.

### Population and eligibility criteria

All healthcare professionals working at public primary hospitals in the Central Gondar zone were the study populations. All health professionals working at Central Gondar zone public primary hospitals during data collection period were included in the study, whereas, health care professionals who had less than six months work experience, critically ill and with annual leave were excluded from the study.

### Sample size determination and sampling procedure

The sample size was determined using single population proportion formula by considering the level of confidence 95%, non-response rate 10%, the margin of error (d = 0.05), and proportion of EBP 53% (31). It was calculated as:


$$\frac{n=Z\;{\left(\alpha /2\right)}^{2*}P\;\left(1-q\right)}{d^{2}}$$

Where, P = 53%; the proportion of EBP utilization.

d = 0.05 (degree of absolute precision).

Z α/2 at 95% confidence level = 1.96.


$$\frac{n={\left(1.96\right)}^{2*}\left(0.53\right)\;\left(1–0.53\right)=383}{{\left(0.05\right)}^{2}}$$

Therefore, by considering 10% non-response rate the final sample size was 422.

**Figure 2:**
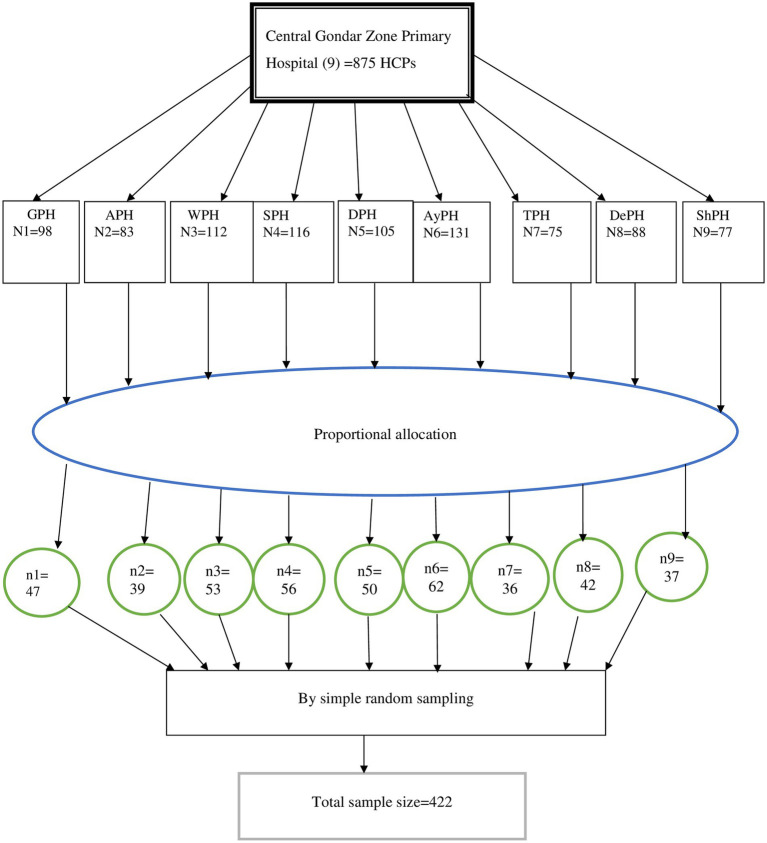
Diagrammatic depiction of sampling procedures, Central Gondar zone public primary hospitals, Northwest Ethiopia, 2022.

There are a total of nine public primary hospitals in such as Guhalla Primary Hospital (GPH), Arbaya primary hospital (APH), Wogera Primary Hospital (WPH), Denbia primary hospital (DPH), Ayikel primary hospital (AyPH), Tegedie primary hospital (TPH), Sanja primary hospital (SPH), Delgi primary hospital (DePH), and Shawra primary hospital (ShPH). To select healthcare professionals (HCPs) from nine primary hospitals in the Central Gondar zone, all hospitals were first listed down with their respective numbers of HCPs and sample size was proportionally allocated to each hospital. Then, the sampling frame was prepared for each hospital by having lists of HCPs from the hospitals’ human resource management. Finally, eligible HCPs of each hospital were selected through simple random sampling technique (Figure 2).

### Variables

#### Outcome variable

EBP was the outcome variable of this study. It was measured through valid and reliable items which were reviewed from previous studies (21, 24, 31, 32). To measure EBP we used the 6 item questions with a five-point Likert scale (never: zero times, rarely: 1–3 times, Sometimes: 4–5 times, often: 6–7 times, and always: 8 or more times), and the options are based on how frequently the respondents perform each of the items over the past 8 weeks.

#### Explanatory variables

Were grouped into sections as socio-demographic variables (sex, age, marital status, educational status, type of profession, years of service and monthly salary), health professionals related variables (presence of smartphone, position status, place of educational completion, knowledge of EBP, attitude toward EBP, self-rated skills to perform EBP), and organizational related variables: availability of internet access, availability of updated guidelines and protocols, relation with nearby boos, training on EBP, its searching strategy and utilization of protocols and guidelines, future stay in their job, organizational support, presence of computer loaded with different reference material, mentor by colleagues who have EBP experience, and availability of spare time for EBP.

### Data collection tools, procedure, and quality assurance

Data were collected using a structured self-administered questionnaire, which is adapted consulting various prior literature (31–34). Nine facilitators, one facilitator per hospital, were assigned and one day training was given on how to collect and facilitate the data, the aim of the study, confidentiality, and the need to take written consent. Every day after data collection, questionnaires were reviewed and checked for completeness. Then, necessary feedback was given to facilitators. The questionnaire was prepared in English and translated into Amharic language and back to English to ensure consistency. The pretest was done on 5% of the sample size at Ebinat primary hospital, to check the response, language clarity, and appropriateness of the questionnaire. Continuous supervision was also made by the principal investigators throughout the data collection period. Finally the tool was checked for reliability (internal consistency) using the Cronbach’s alpha coefficient which was 0.84.

### Operational definitions

#### Good evidence-based practice

Based on the questions designed to measure utilization of EBP, health care professionals who score equal and above 60% were categorized as having “good utilization of EBP” otherwise categorized as “poor utilization of EBP” (33).

#### Knowledge about EBP

##### Knowledgeable

Based on the summative score designed to assess their knowledge level, a score equal and above 80% were considered knowledgeable, otherwise they are considered as ‘not knowledgeable’ (33).

#### Attitude about EBP

##### Favorable attitude

Based on the summative score designed to assess their attitude level, a score of greater than 80% was considered as having a favorable attitude about EBP.

##### Moderate attitude

If participants answered 60–80% of attitude-related questions regarding utilization of EBP were considered as having a moderate attitude about EBP.

##### Unfavorable attitude

If participants answered below 60% of attitude-related questions regarding utilization of EBP were considered an unfavorable attitude about EBP (33).

#### Self-rated skill to perform EBP

##### Adequate skill

A score of >80% was considered as having adequate skill about EBP.

##### Moderate skill

If participants answered 60–80% of skill-related questions regarding the EBP were considered as having moderate skill about EBP.

##### Poor skill

If participants answered below 60% of attitude related questions regarding the EBP were considered as having poor skill about EBP (28, 33).

### Data management and analysis

The data were entered into EpiData version 4.6 and exported to SPSS version 25 for analysis. The descriptive statistical analysis was used to compute frequency and percentages. Bivariable and multivariable binary logistic regression were used to identify factors associated with EBP and to control confounding effects, respectively. Hosmer and Lemeshow test was conducted before the use of this model and it results *p* value of 0.963. First, bivariable logistic regression was done and variables having a *p*-value of <0.25 were eligible to the multivariable logistic regression model to control confounders, and identify the predictors of EBP utilization. Adjusted Odds Ratio (AOR) with 95% confidence intervals and *p*-value <0.05 were used to describe the association and presence of statistical significance.

## Results

### Socio-demographic characteristics of the study participants

A total of 406 health professionals were participated in this study. Of which, 252 (62.1%) were with-in the age category of 26–31 years and with the median age of 29 (SD ± 4.127) years. The largest proportion of the respondents, 279 (68.7%), were males and 229 (56.4%) of them were married.

Regarding the educational level of the respondents, 249 (61.3%) were bachelor degree holders and only 25 (6.2%) of them had master degree. Moreover, over 154 (37.9%) of the respondents were nurses in their profession (Table 1).

**Table 1:** Socio-demographic characteristics of HCPs about utilization of EBP at Central Gondar zone public primary hospitals, Northwest Ethiopia, 2022.

Characteristics category	Frequency	Percentage (%)
**Age**		
20–25	52	12.8
26–31	252	62.1
32–37	79	19.5
>37	23	5.7
**Sex**		
Male	279	68.7
Female	127	31.3
**Type profession**		
Medical doctor	47	11.6
Nurse	154	37.9
Midwifery	55	13.5
Pharmacy	47	11.6
Laboratory	46	11.3
Others*	57	14.1
**Marital status**		
Married	229	56.4
Single	153	37.7
Divorced and Separated	24	5.9
**Educational status**		
Diploma	132	32.5
Degree	249	61.3
Masters and above	25	6.2
**Years of service**		
<5 year	249	61.3
5–10	126	31.1
>10	31	7.6
**Monthly salary**		
<5,000	87	21.4
5,001–7,000	101	24.9
7,001–9,000	135	33.3
>9,000	83	20.4

*Anesthesia, x-ray, psychiatric and integrated emergency surgeon, **Operation room and anti- retro viral therapy room.

### Healthcare professionals-related factors

The majority, 342 (84.2%), of respondents completed their education from government institutions and nearly three-fourth, 293 (72.20%) of the respondents had no leadership role in their hospitals, and 304 (74.9%), of the respondents had smartphones.

**Figure 3:**
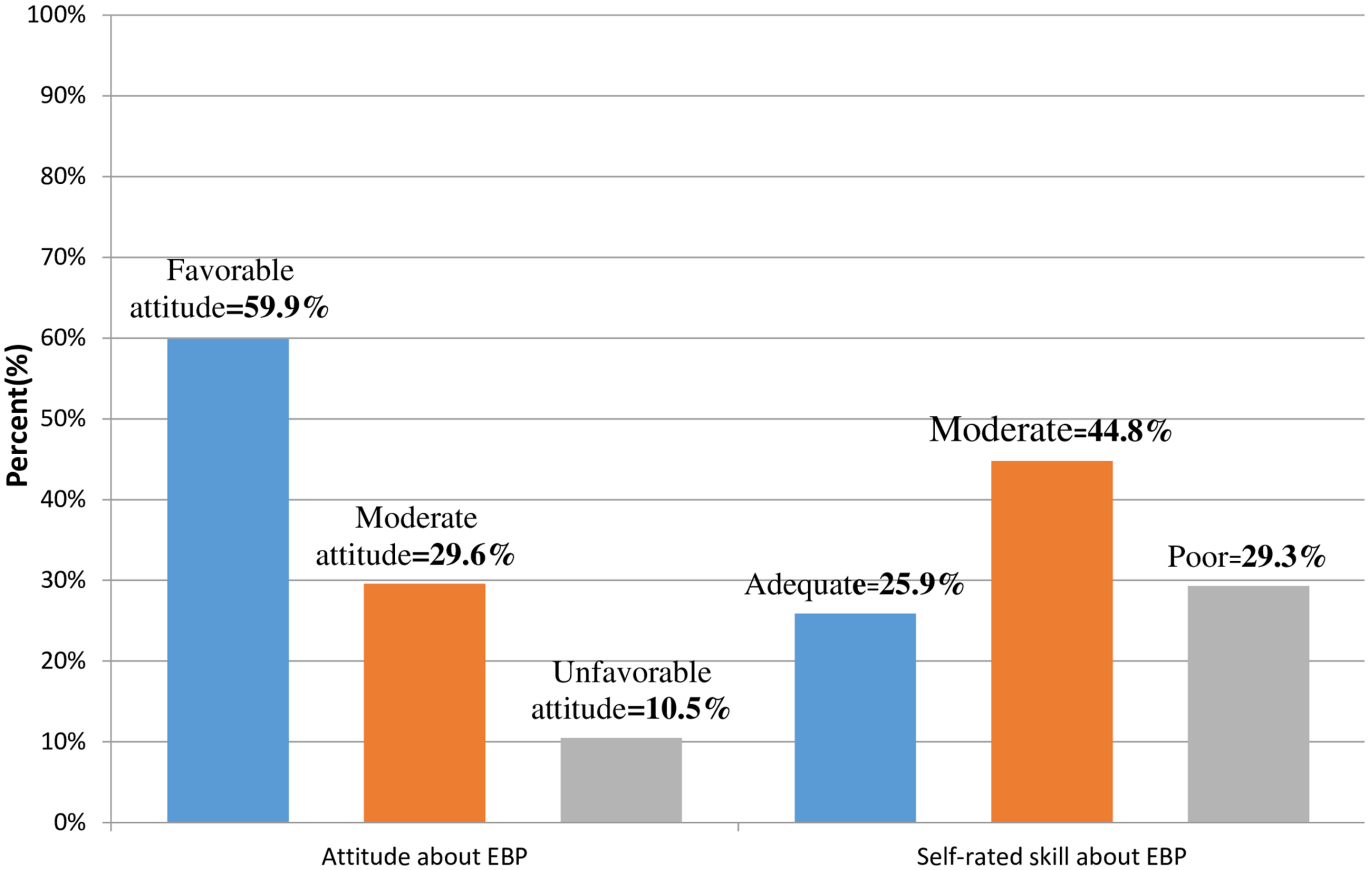
Attitude and self-rated skill about utilization of EBP among HCPs working at central Gondar zone public primary hospitals Amhara region, Northwest Ethiopia, 2022.

Moreover, one-third of the respondents did not ever hear about EBP, 41% (164) of the respondents had no confidence to appraise and judge quality of research, and 135 (33.3%) of the respondents had difficulty understanding research reports. Likewise, 334 (82.2%) of the respondents did not know where published research articles are found. Concerning range of evidence utilization for clinical practices, 59 (14.5%), 157 (38.7%), 135 (33.3%), 273 (67.2%), 185 (45.6%) and 98 (24.1%) were used research findings, books, Google like Medscape and up-to-date, guidelines and protocols, clinical practice observed on daily bases, and consulting seniors, respectively. Over half, 222 (54.7%) of the respondents were knowledgeable about EBP and concerning the attitude and self-rated skill of the respondent, 243 (59.9%) and 105 (25.9%) of them had a favorable attitude and adequate self-rated skill about utilization of EBP, respectively (Figure 3).

### Organizational-related factors

About 226 (55.7%) of the respondents had internet access at work places. Over two-thirds, 159 (39.2%) of the respondents had updated protocols and guidelines at their working place. Nearly 67 and 85% of the respondents did not take training about EBP and searching strategies for finding research evidence for EBP, respectively (Table 2).

**Table 2:** Organizational-related characteristics of HCPs toward utilization of EBP in Central Gondar zone public primary hospitals, Northwest Ethiopia, 2022.

Characteristics	Category	Frequency	Percentage
Organizational support	Yes	190	46.8
	No	216	53.2
Internet access	Yes	226	55.7
	No	180	44.3
Availability of updated guideline and protocols	Yes	159	39.2
	No	247	60.8
Given spare time for EBP	Yes	114	28.1
	No	292	71.9
Mentor by professionals who had EBP experience	Yes	107	26.4
	No	299	73.6
Future intention to stay on their profession	Yes	291	71.7
	No	115	28.3
Training on searching strategy	Yes	63	15.5
	No	343	84.5
Self-rated job satisfaction	Yes	291	71.7
	No	115	28.3
Training about EBP	Yes	135	33.3
	No	271	66.7
Training on guidelines and protocol utilization	Yes	117	28.8
	No	289	71.2
Presence of Computer loaded with different references	Yes	95	23.4
	No	311	76.6

**Figure 4:**
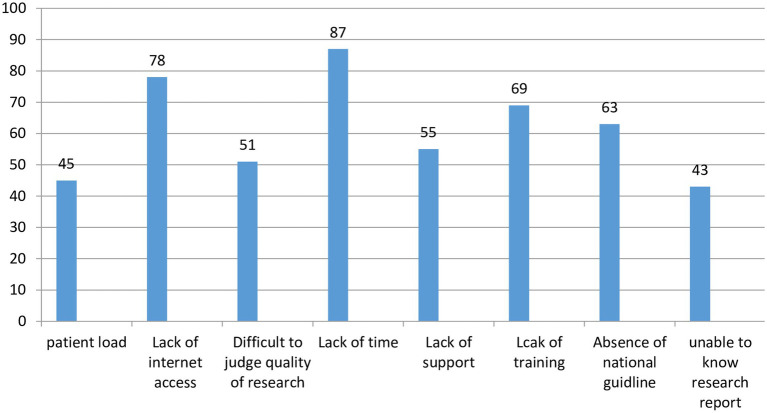
Perceive barriers to utilize EBP among HCPs in Central Gondar zone, Northwest Ethiopia, 2022.

Regarding perceived barriers to utilize EBP were; lack of training about EBP and its searching strategy, absence of good internet access and shortage of time (Figure 4).

### Utilization of evidence-based practice

This study found that the rate of good EBP among healthcare providers working at primary hospitals of the Central Gondar Zone was found to be 44.1% (95% CI: 39, 5%0). About 66 (16.3%) of HCPs sometimes formulate clinical questions. Additionally, 155 (38.2%) of them rarely search evidence for clinical care. Around 18% of the respondents were never critically appraising evidence and one-third of the respondents were rarely applying evidence. Above, three-fourths, 312 (77%) of respondents evaluated the outcomes of their practice, but 89 (22%) of them never shared the outcomes of evidence to their colleagues (Table 3).

**Table 3:** Frequency of EBP utilization among HCPs working at Central Gondar zone public hospitals, Amhara region, Northwest Ethiopia, 2022 (*n* = 406).

Activities of EBP	Never		Rarely		Sometimes		Often		Always	
	*N*	%	*N*	%	*N*	%	*N*	%	*N*	%
Formulating clinical question	145	35.7	174	42.9	66	16.3	17	4.2	4	1.0
Searching best evidences	67	14.0	155	38.2	87	21.4	62	15.3	45	11.1
Critically appraise evidences	71	17.5	140	34.5	91	22.4	70	17.2	34	8.4
Applying best evidence	75	18.5	136	33.5	80	19.7	69	17.0	46	11.3
Evaluate outcome of care	94	23.2	141	34.7	85	20.9	50	12.3	36	8.9
Share outcome to colleagues	89	21.9	143	35.2	87	21.4	55	13.5	32	7.9

### Factors associated with evidence based practice

Bivariable and multivariable logistic regression analysis was done to identify

factors associated with EBP. On the bivariable logistic regression, educational status, marital status, work experience, knowledge about EBP, self-rated skill about EBP, training about EBP, future stay in their profession, organizational support, availability of protocol and guidelines, presence of internet access at work space, spare time given to EBP and self-rated satisfaction about their job of the health care professionals were associated with EBP.

On the multivariable logistic regression analysis, educational status, time given to EBP, presence of internet access, training about EBP, knowledge about EBP, self-rated skill about EBP were significantly associated with EBP.

Healthcare professionals who took training about EBP were 2.1 times more likely to have EBP compared with those who did not take training about EBP [AOR: 2.13, 95% CI (1.26–3.58)]. Healthcare professionals who had good knowledge about EBP were 7.9 times more likely to have good EBP compared with those who had poor knowledge about EBP [AOR: 7.95, 95% CI (4.83–13.07)]. Similarly, health professionals who had relatively enough time to apply EBP were 1.9 times more likely to have good EBP compared to those who did not have relatively enough time to apply EBP [AOR: 1.90, 95% CI (1.09–3.31)]. Health professionals who had internet access were 2 times more likely to utilize EBP compared with those who did not have internet access [AOR: 2.02, 95% CI (1.25, 3.27)]. HCPs who had good self-rated skills about EBP were 2.4 times more likely to utilize EBP compared with those who had poor self-efficacy skills of EBP [AOR: 2.39, 95% CI (1.27–4.53)]. Moreover, HCPs who had masters and above educational level were 3 times more likely to utilize EBP compared with those who had diploma educational status[AOR: 3.05, 95% CI (1.08–8.67)] (Table 4).

**Table 4:** Bivariable and multivariable logistic regression analysis of factors associated with utilization of EBP among HCPs, Central Gondar zone, Northwest Ethiopia, 2022.

Variables	EBP		COR (95%CI)	AOR (95%CI)
	Good	Poor		
Educational status				
Diploma	49	83	1	1
Degree	113	136	1.41 (0.91, 2.17)	1.26 (0.76, 2.11)
Master and above	17	8	3.6 (1.45, 8.96)	3.05 (1.08, 8.67)*
Marital status				
Married	98	131	1	1
Single	67	86	1.07 (0.71, 1.61)	1.11 (0.66, 1.85)
Divorced	5	6	0.76 (0.22, 2.68)	1.30 (0.27, 6.20)
Separated	8	5	3.01 (0.90, 10.05)	1.31 (0.35, 5.05)
Years of service				
<5 year	99	150	1	**1**
5–10 year	62	64	1.46 (0.95, 2.26)	1.21 (0.31, 1.83)
>10 year	18	13	2.09 (0.98, 4.47)	1.15 (0.45, 2.90)
Knowledge about EBP				
Knowledgeable	143	79	7.44 (4.72, 11.74)	7.95 (4.83, 13.08)**
Not Knowledgeable	36	148	1	**1**
Self-rated skill about EBP				
Adequate	65	40	2.87 (1.67, 4.94)	2.39 (1.27, 4.52)*
Moderate	71	111	1.21 (0.74, 1.96)	1.28 (0.73, 2.26)
Poor	43	76	1	1
Trained about EBP				
Yes	77	58	2.20 (1.44, 3.34)	2.12 (1.26, 3.58)*
No	102	169	1	1
Self-rated satisfaction of job				
Satisfied	136	155	1.46 (0.94, 2.28)	1.06 (0.55, 2.02)
Not satisfied	43	72	1	1
Internet access at work place				
Yes	115	111	1.88 (1.26, 2.80)	2.02 (1.25, 3.27)*
No	64	116	1	1
Organizational support				
Yes	100	90	1.93 (1.30, 2.87)	1.42 (0.87, 2.32)
No	79	137	1	1
Given spare time				
Yes	67	47	2.29 (1.47, 3.56)	1.90 (1.09, 3.31)*
No	112	180	1	1
Protocol availability at work place				
Yes	76	83	1.29 (0.86, 1.91)	0.88 (0.52, 1.51)
No	103	144	1	1
Intention to stay for the future				
Yes	138	153	1.63 (1.04, 2.54)	1.39 (0.80, 2.39)
No	41	74	1	1

AOR, Adjusted odd ratio; COR, Crude odd ratio; CI, Confidence interval, ^*^p ≤ 0.05, ^**^ *p* ≤ 0.001. Bold: significant category.

## Discussion

This study aimed to assess EBP and associated factors among HCPs working at Central Gondar zone public hospitals. In this study, the utilization of EBP was found to be 44.1% (95% CI: 39, 50). Educational status, time given to EBP, presence of internet access at work place, training about EBP, knowledge and self-rated skill about EBP were significantly associated with EBP. This finding were comparable with the study done in North West Ethiopia (34), North West Amhara public hospitals (35), Tibebe-Gion, Bahir-Dar, and University of Gondar hospitals (17), and West Amhara (18) that reported 40, 48.4, 47 and 40.8%, respectively.

However, findings of the current study are lower than studies conducted in Black lion hospital (10), Amhara region public hospitals (32), North Gondar (31), and Southern Ethiopia (24) that reported 57.6, 55, 53 and 55% EBP, respectively. The possible reason might be the difference in area they work. This study was conducted among HCPs who work in district level hospitals whereas previous studies were conducted in teaching and referral hospitals. The other probable reason might be the difference in eligibility criteria. The study conducted in Black lion excluded diploma HCPs, but this study includes all HCPs regardless of their educational status, so due to this they may have higher EBP utilization than this study. The other reason may also be the difference in the study population. In this study the study population was all health care professionals but previous studies took nurses.

Likewise this study was also lower than the studies conducted in Uganda 59% (23), Kenya 63.19% (14), Nigeria 78.7% (36). This variation might be due to the difference in educational curriculum, socio-demographic, availability of internet access and training in the organization and the study area.

But this finding was higher as compared to a study conducted in Hararghe zone, 32.3% (37) and Amhara region public hospitals 34.8% (33). This variation might be the difference in sample sizes. Only 137 respondents participated in a study conducted at Hararghe zone and 826 respondents participated in a study conducted at Amhara region public hospitals. The other reason may be the difference in the number and type of items used to measure dependent variables. A study conducted in Amhara region government hospitals used higher items than items used in this study. The other reason may also be the difference in study populations. Study populations of Hararghe zone studies were physicians and study populations of Amhara region public hospitals were nurses and midwifery only, whereas a study population of this study was all health care professionals.

This finding is also higher as compared to a study conducted in Malaysia (38). This variation might be due to the difference in place of work. All Malaysian participants were working in the emergency room only, due-to this; participants may not have a time to utilize EBP.

In this study the odds of utilizing EBP was 3 times higher among HCPs who had an educational level of master as compared to HCPs who had an educational level of diploma. These findings were supported with the study done by Tibebe-Gion and University of Gondar hospitals (17), public hospitals of Addis Ababa (21) and Malawi (3). This is explained by the fact that HCPs who have a master’s degree are more experienced in appraising evidence and more informed about EBP concepts, so; they are more likely to utilize EBP. This implies that HCPs should improve their educational status.

In this study the odds of utilizing EBP was 2.1 times higher as compared with those who did not take training about EBP. This finding was comparable with studies conducted in Amhara region (32), Southern Ethiopia (24) and Saudi university (29). This is because health care professionals who trained about EBP may know the concept of EBP and its utilization strategies. This implies that the responsible body should train HCPs about EBP.

The current study revealed that, the odds of utilizing EBP were 7.9 times higher among HCPs who had good EBP knowledge than those who had poor EBP knowledge. These findings were agreed with the study done in North West Ethiopia (31), Amhara region (32, 33, 35), Southern Ethiopia (24), Jimma zone (4), Nigeria (36) and Uganda (5). This is the fact that knowledge is the prerequisite /entry point to any health care practice. This also implies that to utilize EBP, the knowledge of HCPs should be improved.

Similarly, in this study HCPs who had relatively enough time to apply EBP were 1.9 times more likely to use EBP compared to those who did not have enough time. This finding was supported with the studies conducted in federal public hospital of Addis Ababa (39), Amhara region teaching hospital (35), and hospitals of Northwest Ethiopia (31). This might be the fact that HCPs having insufficient time might face difficulty in searching and reading new evidence.

So, the health care administration should give spare time to HCPs.

This study also found that HCPs who had internet access at work were 2 times more likely to utilize EBP compared with those who did not have internet access. These studies were supported with the study done in Southern Ethiopia (24), North West Ethiopia (31) and Amhara region (32). This is because EBP utilization needs some prerequisites like searching over the internet and easy access to online EBP resources which may simplify the use of evidence-based practice. But this finding is not supported by a study conducted in Malawi, in which availability of internet access has no association with utilization of EBP (3). This might be Malawi HCPs may have personal internet access other than workplace internet access. This implies that health care facility internet access should be improved.

Furthermore, HCPs who had good self-rated skills about EBP were 2.4 times more likely to utilize EBP compared with those who had poor self-rated skills about EBP. This study finding was comparable with the studies conducted in Amhara region hospitals (32, 33). This could be because respondents who had good skill about the application EBP may search evidence through different databases and they know different searching engines to find evidence. And this finding supported that knowledge and skills are recognized as core competency to utilize EBP worldwide (2).

## Limitation of the study

Even if, a better way of understanding was given for the study participants to give factual information, social desirability bias might have also be introduced.

## Conclusion

The rate of good EBP among health professionals working at primary hospitals of the Central Gondar zone is lower compared to national standard. Educational status, the time given to utilize EBP, the availability of internet access, training about EBP, knowledge and self-rated skill about EBP were the determining factors associated with utilization of EBP.

## Data Availability

The original contributions presented in the study are included in the article/supplementary material, further inquiries can be directed to the corresponding author.
